# Cardiopulmonary and hemodynamic responses to *Baduanjin* exercise and cycle ergometer exercise among chronic heart failure patients: a comparison

**DOI:** 10.3389/fphys.2025.1620785

**Published:** 2025-09-18

**Authors:** Xiankun Chen, Xiaoyue Hu, Thomas P. Olson, Yaqi Qiu, Huiying Zhu, Zehuai Wen, Huayang Cai, Weihui Lu, Wei Jiang

**Affiliations:** ^1^ Clinical Research Center, The Second Affiliated Hospital of Guangzhou University of Chinese Medicine, Guangzhou, China; ^2^ The Second Clinical Medical College, Guangzhou University of Chinese Medicine, Guangzhou, China; ^3^ Division of Preventive Cardiology, Department of Cardiovascular Medicine, Mayo Clinic and Foundation, Rochester, MN, United States; ^4^ Department of Cardiology, The Second Affiliated Hospital of Guangzhou University of Chinese Medicine, Guangzhou, China; ^5^ Department of Scientific Research, The Second Affiliated Hospital of Guangzhou University of Chinese Medicine, Guangzhou, China

**Keywords:** chronic heart failure, *Baduanjin*, cycle ergometer exercise, cardiopulmonary response, hemodynamic response

## Abstract

**Objective:**

*Baduanjin* is a traditional Chinese exercise and serves as an alternative to conventional cardiac rehabilitation in China. In this study, we compare the cardiopulmonary and hemodynamic responses of *Baduanjin* to those of cycle ergometer exercise in chronic heart failure patients.

**Methods:**

For this cross-sectional study design, following baseline data collection, participants underwent a series of tests including impedance cardiography (ICG) and a maximal cardiopulmonary exercise test (CPET) to determine peak exercise capacity. Participants then engaged in 9-min of *Baduanjin* exercise. The average oxygen consumption (EqualVO_2_) during *Baduanjin* was calculated. Participants then engaged 9 min of constant-load cycling at 60 rpm at an intensity which elicited the EqualVO_2_. Cardiopulmonary and hemodynamic data were measured continuously during both Baduanjin and cycling exercise.

**Results:**

A total of 30 participants were included. Although *Baduanjin* and cycling exercise showed similar VO_2_ levels (8.2 ± 1.3 vs. 8.4 ± 1.4, *p* = 0.339, respectively), there was a bimodal distribution during *Baduanjin* exercise compared to a unimodal distribution during cycling exercise. Compared to conventional cycling, *Baduanjin* demonstrated lower respiratory burden which is associated with greater ventilatory efficiency as evidenced by lower respiratory rate values (*p* = 0.003), minute ventilation (*p* < 0.001), end-tidal carbon dioxide pressure (*p* < 0.001), and minute ventilation to carbon dioxide production ratio (*p* < 0.001). In terms of hemodynamic response, *Baduanjin* is demonstrated significantly lower cardiac output (*p* = 0.017) and elevated arterial-venous oxygen difference (*p* = 0.036).

**Conclusion:**

Our study offers novel insight into the cardiopulmonary and hemodynamic differences between *Baduanjin* and cycling when performed at consistent intensity levels. *Baduanjin* demonstrates an intermittent intensity pattern and increased peripheral oxygen utilization, which is attributed to more pronounced muscle activation. Furthermore, *Baduanjin* has been linked to a reduction in both cardiac and respiratory burdens.

## 1 Introduction

Comprehensive exercise-based cardiac rehabilitation (CR) is a Class 1A recommend therapy for patients with chronic heart failure (HF) (Pelliccia et al., 2020; McDonagh et al., 2023; Ponikowski et al., 2016). Patients with HF who engage in exercise based CR demonstrate improved quality of life, reduced hospitalization, and lower mortality rates (Molloy et al., 2024-03; Bozkurt et al., 2021). However, these CR programs are underutilized, with participation rates ranging from 10% to 30% worldwide (Beatty et al., 2023; Ozemek and Squires, 2021; Balady et al., 2011; Grace et al., 2008-10; Sanderson et al., 2003). In China, primary barriers to participation in CR have been identified and include resource scarcity and limited healthcare funding (Zhang et al., 2023; Zhang et al.). Therefore, solutions to improving CR uptake HF patients must be tailored to these barriers. In addition to overcoming the above noted barriers, resource-adapted CR programs must be sensitive to the cultural context in which they are embedded.

Commonly accepted as beneficial to one’s health, traditional Chinese exercise is a form of exercise embedded within communities throughout different regions of mainland China for nearly sixteen centuries (Wang et al., 2016; Health Qigong Management Center, 2003). Thus, this equipment-free exercise may be ideal for hospitals with limited resources (Sun, 2015), as well as patients, because it can be practiced at home, reducing barriers such as weather, transportation, and cost (Deka et al., 2017; Conraads et al., 2012). *Baduanjin*, translated as “eight silken movements”, is one form of traditional Chinese exercise which has been practiced for over 1,000 years. It is characterized by slow movements (physical training) synchronized with meditation (mindfulness-based training) and regulated breathing (respiratory training) to achieve a harmonious flow of energy (*qi*) in the body (Zou et al., 2017). *Baduanjin* is easy to learn, with minimal physical or cognitive demands, as it only entails eight simple movements based on traditional Chinese medicine theory. Moreover, it is an adaptable form of exercise that can be practiced in any location, at any time, without any special equipment, and requires minimal time investment (Chen et al., 2022). Hence, it is easily incorporated into daily routines and could easily be integrated into a comprehensive CR program.

Moderate intensity continuous aerobic exercise is the most widely researched type of exercise in CR and has been shown to be efficient, safe, and well-tolerated by patients with HF (Molloy et al., 2024-03). Historically, *Baduanjin* has been considered light-intensity exercise, regardless of the individual’s physical fitness. In contrast, our group has recently demonstrated that *Baduanjin* exercise falls within the moderate intensity aerobic exercise classification, based on the percent of measured oxygen consumption (VO_2_), particularly for deconditioned patients such as those with HF (Chen et al., 2020). However, to date there is a lack of evidence comparing *Baduanjin* with conventional moderate intensity aerobic exercise such as cycle ergometer exercise, among CHF patients. This highlights a key knowledge gap for referring physicians. Therefore, the purpose of this study was to 1) compare the cardiopulmonary responses between *Baduanjin* and cycle ergometer exercise; and 2) compare the hemodynamic responses between *Baduanjin* and cycle ergometer exercise, in patients with CHF.

## 2 Methods

### 2.1 Participants

We utilized a cross-sectional design to recruit patients from the chronic disease management cohort at the Heart Failure Center at Guangdong Provincial Hospital of Chinese Medicine, between June 2023 and January 2024. Eligible participants included stable heart failure patients aged 18–85 years, classified as New York Heart Association (NYHA) class II or III, who had practiced *Baduanjin* for at least 3 months. The exclusion criteria for the trial included conditions that contraindicate exercise testing. Inclusion and exclusion criteria details are listed in Supplementary S1.

### 2.2 Data collection

#### 2.2.1 Procedures, equipment, and requirements

**FIGURE 1:**
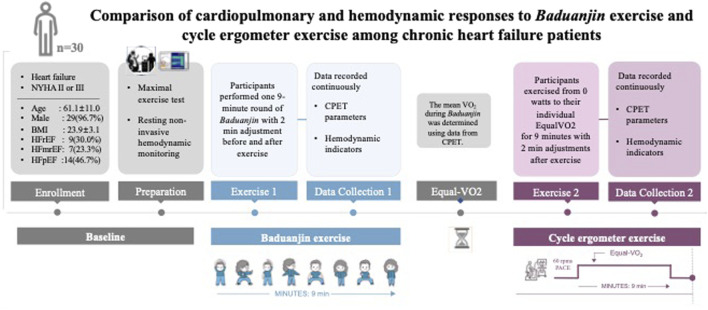
Study procedures Legends: NYHA, New York Heart Association; BMI, body mass index; HFrEF, heart failure with reduced ejection fraction; HFmrEF, heart failure with middle ranged ejection fraction; HFpEF, heart failure with perceived ejection fraction; VO_2_, volume of Oxygen; EqualVO2, the average oxygen consumption; CPET, cardiopulmonary exercise testing.

Our study was conducted in the CR department at the Heart Failure Center at Guangdong Provincial Hospital of Chinese Medicine. The main process of this study included three steps (Figure 1). First, after collecting the baseline information (i.e., medical history, physical examination, anthropometric measurements, and echocardiograph data), we conducted a maximal cardiopulmonary exercise test to determine individual maximal exercise capacity (e.g., VO_2_ peak). Second, we conducted a real-time monitoring test of cardiopulmonary and hemodynamic metrics during *Baduanjin* exercise. Third, we conducted a real-time monitoring test of the same cardiopulmonary and hemodynamic metrics during cycle ergometer exercise. Details of data collection were listed in Supplementary S2. All of the hemodynamic data collected and constructed by PhysioFlow are listed below with their clinical meanings listed in Table 1.

### 2.3 Statistical analysis

We adopted statistical analyses similar to those described in our previous work (Chen et al., 2020). Data from the maximal exercise test as well as the average cardiorespiratory and hemodynamic parameters obtained during *Baduanjin* exercise and cycle ergometer exercise were summarized as mean and standard deviation (SD). The mean VO_2_ and HR collected during *Baduanjin* exercise and cycle ergometer exercise were compared to individual maximum exercise capacity measured during the cardiopulmonary exercise test and reported as a percentage (expressed as %VO_2max_ and %HR_max_). In order to categorize the exercise intensity, we referred to a position statement on exercise intensity terminology (Norton et al., 2010). Moreover, in order to explore the cardiopulmonary response patterns throughout the session, all cardiopulmonary parameters were summarized at 10-s intervals as mean ± SD, and plotted over time. All statistical procedures were performed with SPSS (version 18.0, Chicago, IL, USA).

## 3 Results

### 3.1 Participant characteristics

The participants (n = 30, 29 male and 1 female, ages 61.1 ± 11.0 years) had a clinical history of heart failure for a median of 3.5 years and were classified as either New York Heart Association (NYHA) II (n = 19, 63.3%) or NYHA III (n = 11, 36.7%). The mean left ventricular ejection fraction (LVEF) was 49.4% ± 13.4%, and they were either with reduced LVEF (HFrEF, n = 9, 30.0%), middle ranged LVEF (HFmrEF, n = 7, 23.3%), or preserved LVEF n (HFpEF, n = 14, 46.7%). In addition, 96.7% of the participants (n = 29) had been taking β-blockers. Spirometry showed that 66.7% (n = 20) of participants had normal lung function; and 26.7% (n = 8), 3.3% (n = 1), and 3.3% (n = 1) showed restrictive, obstructive, and mixed pulmonary ventilatory dysfunction, respectively. Details on demographic and anthropometry characteristics, medical history, heart failure status, and comorbidities are presented in Table 2.

**TABLE 1:** Cardiopulmonary and hemodynamic parameters collected in this study.

A. Hemodynamic parameters			
	Parameters	Abbreviations	Clinical meaning
Left Ventricular Ejection Function	Stroke Volume	SV (mL)	The amount of blood pumped out by the heart with each contraction
	Cardiac Output	CO (L/min)	The amount of blood the heart pumps per minute, CO = SV * HR
Myocardial Contraction	Contractility Index	CI	An index to evaluate heart contraction function
Preload	Early Diastolic Filling Rate	EDFR (%)	The rate of left ventricular filling in the early diastolic phase
Afterload	Systemic Vascular Resistance	SVR (dyn∙s/cm^5^)	The systemic peripheral vessels’ resistance to cardiac pumping
Peripheral oxygen utilization	Arterial-venous oxygen difference	C_(a-v)_O_2_ (mL/dL)	The difference in oxygen content between arterial and venous blood, reflecting the balance between oxygen delivery and consumption at the tissue level
B. Cardiopulmonary parameters			
Exercise Tolerance	Load	Load (W)	The load imposed on the body during exercise
	Maximum Oxygen Consumption	VO_2max_ (mL/kg/min)	The amount of oxygen consumed per minute during maximum intensity exercise
	Anaerobic Threshold	AT	The critical point at which anaerobic metabolism exceeds aerobic metabolism during exercise
	Respiratory Exchange Ratio	RER	The ratio of carbon dioxide production to oxygen consumption during respiration
	Metabolic Equivalents	METs	The multiple of metabolic rate during exercise compared to the rate at rest, indicating the relative energy metabolism level
Circulatory Function	Heart Rate	HR (bpm)	The number of heart beats per minute
	Maximum Heart Rate	HR_max_ (bpm)	The highest heart rate that can be achieved during maximum exercise intensity
	Oxygen Pulse	O_2pulse_ (ml/beat)	The amount of oxygen consumed by the body per heartbeat, calculated from VO_2_/HR
	Systolic Blood Pressure	SBP (mmHg)	Blood pressure value during heart contraction
	Diastolic Blood Pressure	DBP (mmHg)	Blood pressure value during heart relaxation
	1-min Heart Rate Recovery	HRR_1_ (bpm)	An index of parasympathetic activity
Ventilation Function	Minute Ventilation	V_E_ (L/min)	The amount of gas exhaled per minute
	Respiratory Rate	RR (bpm)	The number of breaths per minute
Gas Exchange	End-Tidal Carbon Dioxide Pressure	P_ET_CO_2_ (mmHg)	The carbon dioxide pressure in the terminal airways during exhalation
	Ventilation/Carbon Dioxide Production	V_E_/VCO_2_	The ratio of ventilation per carbon dioxide production
	Ventilation/Carbon Dioxide Production Slope	V_E_/VCO_2_ slope	The rate of increase in ventilation per unit increase in carbon dioxide production

**TABLE 2:** Participants’ characteristics (n = 30).

Parameters	Mean ± SD or number (%) (n = 30)
Demographic and anthropometrical characteristics	
Male	29 (96.7%)
Age, years	61.1 ± 11.0
BMI	23.9 ± 3.1
Smoker	10 (33.3%)
Drinker	5 (16.7%)
Heart failure characteristics	
Heart failure history, years	3.5 (2.0, 6.0) ^▲^
NYHA classification	
- NYHA II	19 (63.3%)
- NYHA III	11 (36.7%)
LVEF classification	
-HFrEF	9 (30.0%)
-HFmrEF	7 (23.3%)
-HFpEF	14 (46.7%)
HR, bpm*	67.7 ± 10.8
SBP, mmHg*	108.8 ± 14.1
DBP, mmHg*	66.8 ± 9.8
NT-proBNP, pg/mL	394.0 (189.5,855.1) ^▲^
LVEF, %	49.4 ± 13.4
PASP, mmHg	25.2 ± 5.9
β-blocker users	29 (96.7%)
Comorbidities	
Coronary heart disease	21 (70.0%)
Previous MI	12 (40.0%)
Atrial fibrillation	6 (20.0%)
Hypertension	14 (46.7%)
Type 2 diabetes	14 (46.7%)
Chronic kidney disease	7 (23.3%)
Peripheral vascular disease	14 (46.7%)
Lung function	
FVC(%)	84.2 ± 11.7
FEV_1_ (%)	84.9 ± 10.5
FEV_1_/FVC(%)	105.2 ± 7.4
MVV(%)	96.6 ± 17.5

^▲^ *Median* (*P*_25_, *P*_75_).

*Collected by the research nurse using an electronic sphygmomanometer as the baseline information.

Abbreviations: bpm, beats per minute; BMI, body mass index; DBP, diastolic blood pressure; EF, ejection fraction; FEV1, forced expiratory volume in 1 s; FVC, forced vital capacity; HFrEF, heart failure with reduced ejection fraction; HFmrEF, heart failure with middle ranged ejection fraction; HFpEF, heart failure with perceived ejection fraction; HR, heart rate; LVEF, left ventricular ejection fraction; MI, myocardial infarction; MVV, maximum voluntary ventilation; NYHA, new york heart association; NT-proBNP, N-terminal B-type natriuretic peptide; PASP, pulmonary artery systolic pressure; SBP, systolic blood pressure; SD, standard deviation.

### 3.2 Results of maximum exercise tests and resting hemodynamic status

As shown in Table 3, the results of the maximum exercise test revealed that the average RER of the 30 participants was 1.1 ± 0.1. All participants stopped testing due to leg muscle fatigue. VT was detected in all cases. Participants had an impaired exercise capacity with a mean VO_2max_ of 18.4 ± 4.2 mL/kg/min and demonstrated an elevated V_E_/VCO_2_ slope (30.8 ± 5.8).

**TABLE 3:** Ventilation and gas exchange data during maximal exercise and resting hemodynamic data.

Parameters	Values (Mean ± SD)
Load (W)_MAX	91.7 ± 30.4
A. Ventilation and Gas Exchange during maximal exercise	
Maximum RER	1.1 ± 0.1
Maximum VO_2_ (mL/kg/min)	18.4 ± 4.2
Maximum METS	5.2 ± 1.2
Maximum RR (bpm)	33.4 ± 5.0
Maximum V_E_ (L/min)	48.1 ± 10.4
Maximum P_ET_CO_2_ (mmHg)	37.4 ± 5.6
VE/VCO_2_ slope^△^	30.8 ± 5.8
B. Hemodynamics during rest	
Resting HR (bpm)*	70.2 ± 8.2
Resting SV (mL)	76.6 ± 14.4
Resting CO (L/min)	5.2 ± 1.1
Resting CI	152.8 ± 54.6
Resting O_2_ pulse (mL/beat)	4.3 ± 1
Resting EDFR (%)	63.6 (49.4, 71.4)^▲^
Resting SBP (mmHg)*	117.3 ± 22
Resting DBP (mmHg)*	73.3 ± 17.8
Resting SVR (dyn∙s/cm^5^)	2202.0 (1948.0, 2376.0)^▲^
Resting C_(a-v)_O_2_ (mL/dL)	5.18 ± 2.2

^▲^ *Median* (*P*_25_, *P*_75_).

^△^from rest to maximum.

*Collected during the “Maximal exercise test”.

Abbreviations: bpm, beats per minute; CI, contractility index; CO, cardiac output; C_(a-v)_O_2_, arterial-venous oxygen difference; DBP, diastolic blood pressure; EDFR, early diastolic filling rate; HR, heart rate; MAX, maximal intensity; METs, metabolic equivalents; O_2_ pulse, oxygen pulse (oxygen consumption to heart rate ratio); P_ET_CO_2_, end-tidal carbon dioxide partial pressure; RR, respiratory rate; RER, respiratory exchange ratio; SBP, systolic blood pressure; SD, standard deviation; SV, stroke volume; SVR, systemic vascular resistance; V_E_, minute ventilation; V_E_/VCO_2_ slope, ventilation efficiency for carbon dioxide elimination; VO_2_, oxygen consumption.

The results of hemodynamic assessment indicated that this sample exhibited normal resting SV at 76.6 ± 14.4 mL, CO at 5.2 ± 1.1 L/min, C_(a-v)_O_2_ at 5.18 ± 2.2 mL/dL, and SVR with a median of 2,202.0 dyn∙s/cm^5^. Additional details regarding hemodynamic indices are presented in Table 3.

### 3.3 Comparison of the average cardiopulmonary and hemodynamic responses between *Baduanjin* exercise and cycle ergometer exercise

For the average cardiopulmonary responses (Table 4), the intensity of the two exercises are similar. During exercise, the average VO_2_ during *Baduanjin* was 44.6% of VO_2max_ compared to 45.7% of VO_2max_ during cycling with no statistically significant difference between the two groups (8.2 ± 1.3 vs. 8.4 ± 1.4, *p* = 0.339, respectively; Table 4). For ventilatory and metabolic measures, *Baduanjin* demonstrated significantly lower RR, V_E_, P_ET_CO_2_, and V_E/_VCO_2_ (all *P* < 0.001), compared to the cycling exercise (Table 4).

**TABLE 4:** Comparison of cardiopulmonary and hemodynamic responses to *Baduanjin* exercise or cycle exercise.

Parameters	*Baduanjin* (Mean ± SD)	Cycling (Mean ± SD)	*P*-value
Load (W)	—	21.9 ± 8.4	-
A. Ventilation and Gas Exchange			
VO_2_ (mL/kg/min)	8.2 ± 1.3	8.4 ± 1.4	0.339
METs	2.3 ± 0.4	2.4 ± 0.4	0.299
RR (bpm)	21.1 ± 4.6	23.2 ± 3.3	0.003*
V_E_ (L/min)	18.8 ± 4.5	21.3 ± 3.9	<0.001*
P_ET_CO_2_ (mmHg)	31.6 ± 3.2	34.0 ± 3.4	<0.001*
V_E/_VCO_2_	39.5 ± 5.4	42.0 ± 5.4	<0.001*
B. Hemodynamics			
HR (bpm)	81.8 ± 9.7	79.9 ± 10.2	0.005*
HR_max_ (bpm)	93.9 ± 12.0	86.0 ± 11.9	<0.001*
HRR_1_ (bpm)	5.0 (2.0, 8.0)^▲^	5.0 (3.0, 8.0)^▲^	0.343
SV (mL)	64.5 ± 11.3	70.9 ± 10.4	0.001*
CO (L/min)	5.2 ± 0.8	5.6 ± 0.9	0.017*
CI	145.9 ± 57.8	176.9 ± 63.4	<0.001*
O_2_ pulse (ml/beat)	6.8 ± 1.5	7.2 ± 1.4	0.001*
EDFR (%)	60.1 (56.4, 68.2)^▲^	59.5 (53.1, 69.6)^▲^	0.586
SBP (mmHg)	109.1 ± 16.4	113.1 ± 15.7	0.021*
DBP (mmHg)	65.9 ± 8.1	65.9 ± 10.1	0.544
SVR (dyn∙s/cm^5^)	1,451.4 (1,198.7, 1663.7)^▲^	1,140.1 (1,040.9, 1220.9)^▲^	<0.001*
C_(a-v)_O_2_ (mL/dL)	11.2 ± 3.5	10.4 ± 2.6	0.036*

^▲^ *Median* (*P*_25_,*P*_75_).

Abbreviations: bpm, beats per minute; C_(a-v)_O_2_, arterial-venous oxygen difference; CI, contractility index; CO, cardiac output; DBP, diastolic blood pressure; EDFR, early-diastolic filling rate; HR, heart rate; HR_max_, maximum heart rate during exercise; HRR_1_, 1-min heart rate recovery; METs, metabolic equivalents; O_2_ pulse, oxygen pulse (oxygen consumption to heart rate ratio); P_ET_CO_2_, end-tidal carbon dioxide partial pressure; RR, respiratory rate; SBP, systolic blood pressure; SD, standard deviation; SV, stroke volume; SVR, systemic vascular resistance; V_E_, minute ventilation; V_E_/VCO_2_ slope, ventilation efficiency for carbon dioxide elimination; VO_2_, oxygen consumption.

For the average hemodynamic responses (Table 4), the average HR during *Baduanjin* exercise was significantly higher when compared to cycling (Table 4). Similarly, the peak heart rate during *Baduanjin* was significantly higher than during cycle exercise (93.9 bpm *versus* 86.0 bpm, *P* < 0.001, Table 4). Furthermore, our data indicate that SBP was significantly lower during *Baduanjin* compared to cycling while DBP was not different between the two activities (Table 4). In addition, the average SV and CO during *Baduanjin* exercise (SV: 64.5 ± 11.3 mL, CO: 5.2 ± 0.8 L/min) were both significantly lower than cycle exercise (SV: 70.9 ± 10.4 mL, *P* = 0.001; CO: 5.6 ± 0.9 L/min, *P* = 0.017). Moreover, significantly higher C_(a-v)_O_2_ (*P* = 0.036) and SVR (*P* = 0.036) were found during *Baduanjin* exercise than during cycle exercise.

### 3.4 Comparison of the real-time cardiopulmonary and hemodynamic responses between *Baduanjin* exercise and cycle ergometer exercise

**FIGURE 2:**
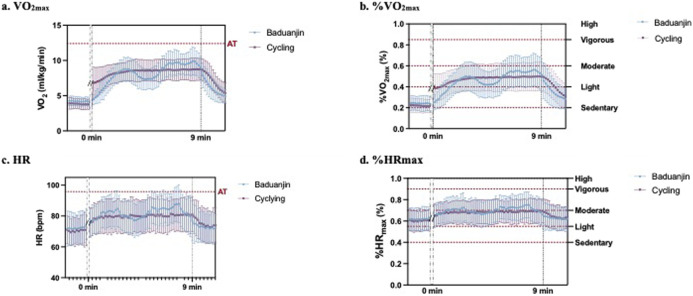
Comparison of the real-time cardiopulmonary responses of **(a)** VO_2_max, **(b)** %VO_2_max, **(c)** HR, and **(d)** %HRmax between Baduanjin exercise and cycle ergometer exercise.

The VO_2_ responses are shown in Figures 2a,b. The VO_2_ response curve of the cycle exercise is smooth with no obvious peaks. After the cycle ergometer exercise reached moderate intensity in the second minute, it leveled off and remained at moderate intensity. However, the fluctuations in VO_2_ during the *Baduanjin* exercise are greater than those during the cycle exercise. The VO_2_ responses exhibited a bimodal distribution during *Baduanjin*. The VO_2_ increased during the first 3 min when a hemi-squat posture was involved (second posture), followed by a small drop in VO_2_ after the transition to third posture. The intensity reached the second peak at the seventh minute during an additional two hemi-squat postures (fifth and seventh). It then dropped to the baseline during the resting phase. Overall, the absolute VO_2_ response remained under the VT (Figure 2a) and the %VO_2max_ response remained within the moderate-intensity range (Figure 2b).

The HR responses are shown in Figures 2c,d. Similar to the VO2 response, the HR response was smooth during cycle exercise and exhibited a bimodal distribution with smaller magnitudes during *Baduanjin* (Figures 2c,d). Overall, the absolute HR response remained under the equivalent HR for the VT (Figure 2c) and the %HRmax response remained within the moderate-intensity range (Figure 2d).

**FIGURE 3:**
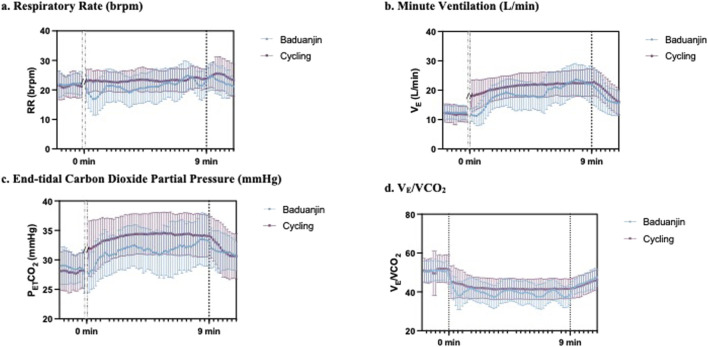
Comparison of the real-time pulmonary responses of **(a)** respiratory rate, **(b)** minute ventilation, **(c)** end-tidal carbon dioxide partial pressure, and **(d)** V_E_/VCO_2_ between Baduanjin exercise and cycle ergometer exercise.

Figure 3 shows the participants’ ventilatory responses, including respiratory rate (Figure 3a), V_E_ (Figure 3b), P_ET_CO_2_ (Figure 3c), and V_E_/VCO_2_ (Figure 3d), during *Baduanjin* and cycling exercise. All three response lines from *Baduanjin* were lower than those from cycling.

**FIGURE 4:**
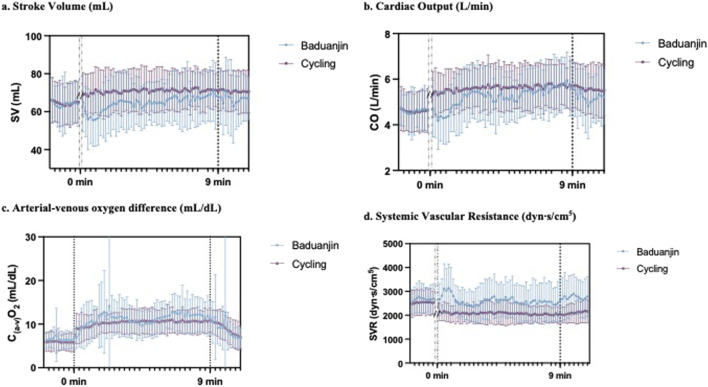
Comparison of the real-time hemodynamic responses of **(a)** stroke volume, **(b)** cardiac output, **(c)** arterial-venous oxygen difference, and **(d)** systemic vascular resistance between Baduanjin exercise and cycle ergometer exercise.

Figure 4 illustrates the real-time hemodynamic responses during *Baduanjin* and cycle exercise. Overall, during cycling, hemodynamics initially increased from a low value and then stabilized, while *Baduanjin*’s SV and CO response lines showed fluctuations with time and movement changes. Compared to cycle exercise, the SV and CO response were both lower during *Baduanjin* (Figures 4a,b), while the C_(a-v)_O_2_ and SVR response was higher during *Baduanjin* (Figures 4c,d).

## 4 Discussion

This is the first study to compare chronic heart failure patients’ cardiopulmonary and hemodynamic responses to *Baduanjin* exercise to their responses to constant-load moderate intensity cycle exercise. The intensity of both exercise modalities was calibrated according to VO_2_, resulting in comparable average VO_2_ for each modality. In terms of the cardiopulmonary response, *Baduanjin* exercise is characterized by a bimodal distribution of VO_2_ responses as well as a lower respiratory burden, which is associated with greater ventilatory efficiency compared to conventional cycling. In terms of hemodynamic response, *Baduanjin* is demonstrated significantly lower cardiac output and elevated arterial-venous oxygen difference. These cardiopulmonary and hemodynamic responses to *Baduanjin* may be attributed to a higher degree of muscle engagement compared to cycle ergometer exercise.

The strength of this study lies in its comparison of the cardiopulmonary and hemodynamic differences between *Baduanjin* exercise and cycle exercise during matched intensity levels. Our study suggests that *Baduanjin* is a moderate-intensity aerobic exercise suitable for CHF patients, similar to our prior findings (Chen et al., 2020). However, there is a lack of research on real-time physiological responses comparing these two exercise modalities. When studying the cardiopulmonary and hemodynamic responses to different exercises, maintaining a consistent intensity level is crucial (Taylor et al., 2021). In a previous study, exercise intensity was matched based on heart rate response (Gary et al., 2019), while in this study, mean VO_2_ during *Baduanjin* exercise was used for match intensity. As such, there was no significant difference in average VO_2_ between the two modes of exercise. This suggests that VO_2_ as a parameter is a good tool for balancing determining exercise intensity as complexities may arise when using HR due to the HR-modulating effect of pharmacotherapies commonly prescribed for CHF patients, such as β-blockers.

While average VO_2_ levels were comparable between the two modes of exercise, distinct differences emerged in the intensity response curves. Cycle exercise plateaued in intensity due to a consistent power output, whereas *Baduanjin* exhibited an intermittent pattern with notable fluctuations, leading to a bimodal response curve. The bimodal VO_2_ response observed during *Baduanjin* exercise is a reflection of the unique characteristics of this traditional Chinese practice, likely attributed to its structured sequence of movements with varying intensities. The presence of two VO_2_ peaks corresponds to movements involving semi-squat postures, indicating that *Baduanjin* offers a distinctive form of moderate-intensity intermittent training. Moderate-intensity intermittent physical activity has been shown to be associated with improved executive function in older adults (MacDonald et al., 2024). Furthermore, a systematic review demonstrated that following training, moderate-intensity intermittent training results in greater reductions in fat mass, as well as improved performance on functional tests for elderly women, compared to moderate-intensity continuous training (Coswig et al., 2020/02). Therefore, *Baduanjin* exercise may be particularly suitable for heart failure patients with impaired executive function or functional performance, as well as for those aiming to achieve fat loss.

*Baduanjin* exercise imposes a lower respiratory burden by enhancing ventilatory efficiency compared to conventional cycle exercise. Similarly, a meta-analysis has also demonstrated that *Baduanjin* improves ventilatory efficiency (Zou et al., 2017). Patients with CHF typically exhibit an exaggerated ventilatory response for a given metabolic demand during exercise (Dubé et al., 2016-09). Our study’s findings indicate that during *Baduanjin* exercise, the respiratory rate is lower than that observed during cycle exercise at the same intensity, suggesting a more stable exercise-induced respiratory response during *Baduanjin*. This can be particularly advantageous for individuals with heart failure working to improve exercise endurance. In addition, our study show that both V_E_ and V_E_/VCO_2_ were lower during *Baduanjin* exercise, compared to cycle exercise. V_E_ quantifies the total volume of gas inhaled or exhaled per minute and is influenced by respiratory rate and tidal volume. The V_E_/VCO_2_ ratio indicates the proportion of ventilation relative to carbon dioxide production. The observed decrease in V_E_ and V_E_/VCO_2_ during *Baduanjin* exercise suggests that less ventilation is needed for the same amount of carbon dioxide production. This indicates a higher efficiency for gas exchange per breath compared to cycle exercise. *Baduanjin* exercise involves movements that elongate respiratory muscles, enhance thoracic compliance and mobility, reduce respiratory center stimulation, and reduce exertional dyspnea (Xie et al., 2022). Additionally, it incorporates respiratory muscle and breathing training to increase respiratory muscle strength and endurance, decrease mechanical loads such as chest wall stiffness, and facilitate deeper, slower breathing for improved gas exchange efficiency.

*Baduanjin* exercise imposes a lower cardiac demand with increased peripheral oxygen utilization, compared to cycling. Our results demonstrate a significantly lower CO response during *Baduanjin* compared with cycling. This reduction in CO, which represents the volume of blood the heart pumps per minute, suggests that *Baduanjin* imposes less cardiac demand than cycling. Moreover, applying the Fick principle, the C_(a-v)_O_2_ was significantly higher during *Baduanjin* exercise when compared to the cycling. The C_(a-v)_O_2_., which quantifies the oxygen concentration disparity between arterial and venous blood post-circulation through active muscle, indicates the efficiency with which peripheral organs, tissues, and cells extract oxygen from the mitochondria. Thus, patients engaged in *Baduanjin* exercise may experience improved peripheral oxygen extraction, potentially due to the engagement of multiple muscle groups characteristic of this form of exercise.

*Baduanjin* has been recognized for its comprehensive muscle training program which targets both the upper and lower extremities. In contrast to cycle exercise, which involves simple movements with less muscle mass engagement, *Baduanjin* incorporates a diverse range of movements that engage muscles throughout the body. For instance, postures such as the horse-riding stance in Postures 2, 5, and 7 can be likened to targeted quadriceps training, effectively strengthening the thigh muscles. Additionally, dynamic movements involving the forearms and fists in Postures 1, 2, 3, and 7 provide comprehensive upper extremity training, enhancing strength and coordination in the arms and hands. Furthermore, *Baduanjin* emphasizes high-impact and weight-bearing exercises, as illustrated in Posture 8 where practitioners push upward from their toes and land forcefully on their feet. Thus, this holistic approach to physical conditioning extends beyond the focus on lower body muscle endurance typically associated with cycle exercise. However, our current data do not allow us to distinguish perfusion changes specifically in the upper or lower extremities. The higher SVR observed during *Baduanjin* compared to that during cycle ergometer exercise may suggest greater muscular engagement, further evidenced by high C_(a-v)_O_2_. Previous research demonstrated that a single bout of resistance training elevates systemic peripheral resistance (Wakeham et al., 2025a; Wakeham et al., 2025b; Dawson et al., 1985; Miles et al., 1987). Therefore, the engagement of multiple muscle groups in *Baduanjin* contributes to its therapeutic benefits, particularly in expanding practitioners’ functional capacity and overall muscle strength (Esposito et al., 2011).

### 4.1 Study limitations

As with any study, this study has potential limitations. Firstly, the sample size was small and we were unable to perform a sample size calculation, as we did not find adequate data for our research question and study design *a priori*. Although the number of participants was low, the within-subject repeats narrowed the estimates’ confidence intervals. Secondly, the interpretability and generalizability of the results are limited by the analyzed population’s clinical characteristics; our study population included mainly NYHA II CHF patients, and only one female. Therefore, our findings are specific to the population studied. Thirdly, our research employed a non-invasive technique known as ICG, which offers benefits for patients. However, ICG also has limitations regarding specific hemodynamic measurements (such as volume and contractility) that need to be estimated or recalculated. Fourthly, it is crucial to recognize that *Baduanjin* exercise encompasses elements of strength training and balance training in addition to endurance exercise training. This multifaceted nature sets *Baduanjin* apart from endurance cycling, which primarily focuses on cardiovascular endurance. As such, the unique combination of strength, balance, and endurance components in *Baduanjin* requires careful consideration when interpreting the results and comparing it to other forms of exercise. Fifth, this study was specifically designed to observe the gas and hemodynamic changes during the two types of exercise. We did not measure the changes in peak VO_2_ or VO_2_ at the first ventilatory threshold following the two types of exercise in this study. However, these parameters are crucial for reflecting improvements in endurance capacity (Christou et al., 2024). Comparing these two parameters would be valuable for further elucidating the differences between the two exercises.

## 5 Conclusion

Our study offers novel insight into the cardiopulmonary and hemodynamic differences between *Baduanjin* and cycle ergometer exercise when performed at consistent intensity levels. Unlike the steady intensity of cycle exercise, *Baduanjin* exhibits an intermittent intensity pattern which is the result of more prominent muscle activation during various postures. Additionally, *Baduanjin* is associated with superior improvement in oxygen respiratory efficiency and increased peripheral oxygen utilization, which is crucial to CHF patients’ health. Furthermore, *Baduanjin* reduces cardiac and respiratory burden, providing a more comfortable exercise experience. From the clinical perspective, the practice’s ease-of-use and flexibility regarding time commitment and space requirements make it an attractive option for cardiac rehabilitation programs. Given these advantages, *Baduanjin* would be particularly effective for inclusion in cardiac rehabilitation programs for CHF patients in China, where it is widely practiced and culturally familiar.

## Data Availability

The raw data supporting the conclusions of this article will be made available by the authors, without undue reservation.

## Glossary

CHF: chronic heart failure
ICG: impedance cardiography
CPET: cardiopulmonary exercise testing
NYHA: New York Heart Association
**EqualVO**_**2**_: the average oxygen consumption
**VO**_**2**_: volume of oxygen
**V**_**E**_: minute ventilation
CO: cardiac output
EBCR: exercise-based cardiac rehabilitation
HF: heart failure
HR: heart rate
SV: stroke volume
CI: contractility index
EDFR: diastolic filling rate
SVR: systemic vascular resistance
**VO**_**2**_ **max**: maximum oxygen consumption
RER: respiratory exchange ratio
METs: metabolic equivalents
HRmax: maximum heart rate
**O**_**2**_ **pulse**: oxygen pulse
SBP: systolic blood pressure
DBP: diastolic blood pressure
**HRR**_**1**_: minute heart rate recovery
RR: respiratory rate
**P**_**ET**_ **CO**_**2**_: end-tidal carbon dioxide pressure
**VE/VCO**_**2**_: ventilation/carbon dioxide production
**VE/VCO**_**2**_ **slope**: ventilation/carbon dioxide production slope
VT: ventilatory threshold
AT: anaerobic threshold
**VCO**_**2**_: volume of carbon dioxide
LVEF: left ventricular ejection fraction
SD: standard deviation
**C(a-v)O**_**2**_: elevated arterial-venous oxygen difference
BMI: body mass index
bpm: beats per minute
NT-proBNP: N-terminal B-type natriuretic peptide
PASP: pulmonary artery systolic pressure
MI: myocardial infarction
MAX: maximal intensity
**O**_**2**_ **pulse**: oxygen pulse (oxygen consumption to heart rate ratio)
**FEV**_**1**_: forced expiratory volume in 1 s
FVC: forced vital capacity
MVV: maximum voluntary ventilation
