# Supplementary material for: Cardiopulmonary and hemodynamic responses to Baduanjin exercise and cycle ergometer exercise among chronic heart failure patients: a comparison
Source: Front Physiol. 2025 Sep 18;16:1620785. doi: 10.3389/fphys.2025.1620785 (PMC12488679; doi:10.3389/fphys.2025.1620785)
Supplement: Supplementary file 1 [file Table1.docx]

**Supplementary S1 Inclusion and exclusion criteria**

**Inclusion Criteria**

(1) Between 18-85 years old.

(2) Meet the diagnostic criteria for chronic heart failure.

(3) Classified as NYHA class II or III.

(4) In a stable phase: defined as the chronic heart failure symptoms and signs being stable for over one month.

(5) Willing to provide informed consent and able to cooperate.

**Exclusion Criteria**

(1) Contraindications for exercise testing include the following conditions: within 6 weeks after acute coronary syndrome; life-threatening arrhythmias; acute heart failure (hemodynamic instability); uncontrolled hypertension (systolic blood pressure >200 mmHg and/or diastolic blood pressure >110 mmHg); advanced atrioventricular block; acute myocarditis and pericarditis; moderate to severe aortic stenosis; moderate to severe mitral stenosis; severe aortic or mitral regurgitation; severe obstructive hypertrophic cardiomyopathy; acute systemic disease; intracardiac thrombus; progressive dyspnea at rest or decreased exercise tolerance within the previous 3-5 days; myocardial ischemia with low power exercise load (<2METs, or <50W); uncontrolled diabetes; recent embolism; thrombophlebitis; new-onset atrial fibrillation or flutter.

(2) Situations not suitable for participating in the trial include: currently suffering from severe mental illness, severe respiratory system disorders, renal failure, liver dysfunction, severe hematopoietic system disease, severe neurological and neuromuscular disease, severe metabolic and endocrine system disease, immune function suppression, and any other serious underlying disease; laboratory test indicators exceeding twice the upper limit of the normal reference value, or considered abnormal and unsuitable for the study by the researcher.

(3) Patients with a history of cardiac surgery, cardiac resynchronization, pacemaker treatment within the past 3 months, or who are planning to undergo these procedures during the trial period.

(4) Patients with a history of cardiac arrest within the past year.

(5) Patients diagnosed with primary pulmonary hypertension, peripartum cardiomyopathy or thyroid heart disease.

(6) Patients who have previously been diagnosed with severe osteoporosis, or balance dysfunction leading to a risk of falling during exercise, or who are unable to complete exercise cardiopulmonary testing.

(7) Severe cognitive impairment rendering patients unable to sign informed consent or unable to understand exercise concepts.

(8) Patients currently participating in other clinical trials or planning to participate in other clinical trials during the study period.
