# Supplementary material for: Cardiopulmonary and hemodynamic responses to Baduanjin exercise and cycle ergometer exercise among chronic heart failure patients: a comparison
Source: Front Physiol. 2025 Sep 18;16:1620785. doi: 10.3389/fphys.2025.1620785 (PMC12488679; doi:10.3389/fphys.2025.1620785)
Supplement: Supplementary file 2 [file Table2.docx]

**Supplementary S2 Details of Data collection**

**1. Procedures, equipment, and requirements**

Our study was conducted in the CR department at the Heart Failure Center at Guangdong Provincial Hospital of Chinese Medicine. The main process of this study included three steps (Fig.1). First, after collecting the baseline information (i.e., medical history, physical examination, anthropometric measurements, and echocardiograph data), we conducted a maximal cardiopulmonary exercise test to determine individual maximal exercise capacity (e.g. VO_2_ peak). Second, we conducted a real-time monitoring test of cardiopulmonary and hemodynamic metrics during *Baduanjin* exercise. Third, we conducted a real-time monitoring test of the same cardiopulmonary and hemodynamic metrics during cycle ergometer exercise.

We used an ergospirometry platform (CARDIOVIT, CS-200, SCHILLER, Switzerland) for collection of all cardiopulmonary measurements. Respiratory gas analysis was performed breath-by-breath with a metabolic cart (POWERCUBE, Ganshorn, Germany) and LF8 software (CARDIOVIT, CS-200 ergospirometry, SCHILLER, Switzerland). The maximal exercise test used an electronically calibrated upright bicycle (ERG 911S, SCHILLER, Switzerland).

All cardiorespiratory metrics were recorded on a spirometry platform (Cardiovascular, CS-200, Schiller, Switzerland). Breath-by-breath analysis was performed using a metabolic cart (POWERCUBE, Ganshorn, Germany) and LF8 software (Cardiovascular Systems, CS-200 Spirometry Device, SCHILLER, Switzerland). An electronically calibrated upright bicycle (ERG 911S, SCHILLER, Switzerland) was used in the maximal exercise test. All tests were conducted in a controlled environment laboratory at a temperature of 20-23°C, an atmospheric pressure of 994-1,042 mmHg, and relative humidity of 50-65%. Before each study, the gas analyzer was calibrated at known gas concentrations (6% carbon dioxide [CO_2_] and 15% oxygen [O_2_]), and the flowmeter was calibrated using a 3 L calibration syringe (SCHILLER, Switzerland).

We used impedance cardiography (ICG) (PhysioFlow, Enduro model, Manatec, France) to measure cardiac hemodynamics. This non-invasive technique was found to be valid, accurate, and reproducible at rest and during exercise in both healthy participants and cardiac patients.^1,2^ This device is a portable and non-invasive monitor that utilizes thoracic electrical bioimpedance to estimate cardiac output (CO). It operates by monitoring morphology-based impedance cardiography signals in real-time via a Bluetooth USB adapter, providing wireless connectivity and real-time measurements, which makes it highly suitable for physical activity and/or exercise applications. Placement of the electrodes on the body was conducted according to the method described by Tonelli et al.^3^

Before each test, all participants were asked to rest in a supine position for 30 minutes, while the physician explained the purpose of the test and related precautions. All tests were conducted by the same examiner to prevent any inter-examiner variability. The examiner was both trained in and experienced with all methodologies. All participants were instructed to refrain from eating 2 hours before study visits and to abstain from caffeine, alcohol, and strenuous physical activity on each test day. Water intake was allowed.

**2. Resting non-invasive hemodynamic monitoring**

Before the maximal exercise test, resting hemodynamics were measured via ICG.

After skin preparation with abrasive material (3M, Red Dot, USA) and alcohol tabs before ICG electrode installation we used the recommended ICG electrodes (PhysioFlow PF50 AgCl) to maximize signal quality during ICG acquisition.

Individual recordings with signal quality of at least 70% and within manufacturer recommended range were included. The standard deviation was calculated for each parameter and for each patient and outliers were excluded.

This system allowed for the non-invasive stroke volume (SV), and CO. Additional metrics included contractility index (CI), early diastolic filling rate (EDFR), and systemic vascular resistance (SVR). The arterial-venous oxygen difference (C_(a-v)_O_2_) was is derived according to the Fick principle, expressed as C(a-v)O₂ = VO₂/CO. All of the hemodynamic data collected and constructed by PhysioFlow are listed below with their clinical meanings listed in Table 1.

**3. Maximal exercise test**

Prior to the maximal exercise test, we conducted spirometry according to the guidelines of the American Thoracic Society and European Respiratory Society.^4^ Participants were then instructed to lie down and rest in a supine position for 30-40 minutes before the maximal exercise test. All participants read a standardized script of the testing procedures and received an explanation of the 6–20 Borg Rating of Perceived Exertion scale.

Upon completion of these preliminary procedures, we conducted the maximum exercise test according to the American Thoracic Society/American College of Chest Physicians Statement on Cardiopulmonary Exercise Testing.^5^ This was a symptom-limited exercise test using a bicycle ramp protocol: an initial work rate of 0 watts with 10% predicted maximum work rate increments every minute. Participants were verbally encouraged to maintain the desired pedal rate of 60 rpms and to exercise until exhaustion or fatigue. However, the examiner would stop the test according to established stopping criteria.^6^ Perceived exertion was measured using the 6-20 Borg Scale (every 2 to 3 min) throughout the test.

The exercise test time ranged from 8 to 12 minutes. We used a 12-lead electrocardiogram for continuous measurement of heart rate (HR) and rhythm evaluation. Peripheral oxygen saturation was monitored continuously and systolic blood pressure (SBP) and diastolic blood pressure (DBP) were measured by manual sygmomanometery at rest and every 3 minutes until test termination. Cardiopulmonary parameters for each participant (averaged over 10 seconds) were collected automatically from the system. Cardiopulmonary parameters included: maximum oxygen consumption (VO_2max_), respiratory exchange ratio (RER), metabolic equivalents (METs), oxygen pulse (O_2pulse_), minute ventilation (V_E_), respiratory rate (RR), end-tidal carbon dioxide pressure (PETCO_2_), ventilation/carbon dioxide production (V_E_/VCO_2_), ventilation/carbon dioxide production slope (V_E_/VCO_2 slope_).

The ventilatory threshold (VT) was assessed by the examiner and confirmed by an independent examiner using established criteria.^6^ Both examiners were experienced and certified physicians who received training before the study. If agreement could not be reached, a third physician was invited to discuss, and final VT was determined by consensus. A defined variable list is provided in Table 1.

**4. Baduanjin exercise** **monitoring test**

Following the maximal exercise test, we conducted a *Baduanjin* exercise monitoring test. For the *Baduanjin* monitoring test, we used the standing-form for *Baduanjin* from China’s General Administration of Sport.^7^ Prior to testing, all participants were instructed to rest in a supine position for 30 minutes. Participants then engaged in one round of *Baduanjin* for 9 minutes, following a video with verbal cues on technique. There was a 2-minute breathing adjustment period before and after the practice session. Total test duration was 13 minutes. The same cardiopulmonary data collected during the maximal cardiopulmonary exercise test was collected during the *Baduanjin* exercise session and averaged over 10 seconds. Blood pressure was measured at rest and every 3 minutes until test termination. Cardiac hemodynamics were also measured by ICG during testing.

**5. Cycle ergometer exercise monitoring test**

The mean VO_2_ during *Baduanjin* (EqualVO_2_) was determined using data from the *Baduanjin* exercise monitoring test. The EqualVO_2_ was then used as the prescribed exercise intensity on a cycle ergometer. Following this, we conducted a cycle ergometer exercise monitoring test in which participants exercised from 0 watts to their individual EqualVO_2_. The resistance on the cycle ergometer was titrated to ensure patients reached a steady state at EqualVO_2_. Once a participant reached their EqualVO_2_, they were instructed to maintain a pedal rate of 60 rpms for 9 minutes. The same cardiopulmonary data and cardiac hemodynamics as collected during the *Baduanjin* exercise monitoring test were automatically recorded.

**Reference**

1. Myers J, Prakash M, Froelicher V, Do D, Partington S, Atwood JE. Exercise capacity and mortality among men referred for exercise testing. *N Engl J Med*. Mar 14 2002;346(11):793-801. doi:10.1056/NEJMoa011858

2. Kavanagh T, Mertens DJ, Hamm LF, et al. Prediction of long-term prognosis in 12 169 men referred for cardiac rehabilitation. *Circulation*. Aug 6 2002;106(6):666-71. doi:10.1161/01.cir.0000024413.15949.ed

3. Tonelli AR, Alkukhun L, Arelli V, et al. Value of impedance cardiography during 6-minute walk test in pulmonary hypertension. *Clin Transl Sci*. Dec 2013;6(6):474-80. doi:10.1111/cts.12090

4. Miller MR, Hankinson J, Brusasco V, et al. Standardisation of spirometry. *The European respiratory journal*. Aug 2005;26(2):319-38. doi:10.1183/09031936.05.00034805

5. ATS/ACCP. ATS/ACCP Statement on cardiopulmonary exercise testing. *American journal of respiratory and critical care medicine*. Jan 15 2003;167(2):211-77. doi:10.1164/rccm.167.2.211

6. Wasserman K. *Principles of exercise testing and interpretation : including pathophysiology and clinical applications*. 5th ed. ed. Philadelphia : Wolters Kluwer Health/Lippincott Williams & Wilkins; 2012.

7. GASC. General Administration of Sport of China: Baduanjin. Accessed January 1st, 2019. <http://www.sport.gov.cn/n16/n1107/n1638/3886341.html>

8. Chen X, Marrone G, Olson TP, et al. Intensity level and cardiorespiratory responses to Baduanjin exercise in patients with chronic heart failure. *ESC Heart Failure*. 2020 2020;7(6):3782-3791. doi:<https://doi.org/10.1002/ehf2.12959>

9. Norton K, Norton L, Sadgrove D. Position statement on physical activity and exercise intensity terminology. *Journal of science and medicine in sport*. Sep 2010;13(5):496-502. doi:10.1016/j.jsams.2009.09.008
